# Low-Frequency Noise Evaluation on a Commercial Magnetoimpedance Sensor at Submillihertz Frequencies for Space Magnetic Field Detection

**DOI:** 10.3390/s19224888

**Published:** 2019-11-09

**Authors:** Tao Wang, Chen Kang, Guozhi Chai

**Affiliations:** 1 Key Laboratory for Magnetism and Magnetic Materials of the Ministry of Education, Lanzhou University, Lanzhou 730000, China; twang2015@lzu.edu.cn (T.W.); kangch18@lzu.edu.cn (C.K.); 2 Research Center of Gravitation, Lanzhou University, Lanzhou 730000, China

**Keywords:** low-frequency noise, submillihertz frequencies, magnetoimpedance sensors, space gravitational wave

## Abstract

The purpose of this study was to measure the low-frequency noise and basic performance of a commercial magnetoimpedance (MI) sensor at sub-millihertz frequencies for use in space missions. Normally, space missions require measuring very weak magnetic fields with a long integration time, such as the space gravitational wave detection mission requiring sub-millihertz frequencies. We set up a platform for measuring the performance on this MI sensor, including low-frequency noise, measurement limit, linearity, and temperature stability. The results show that the low-frequency noise of the MI sensor is below 10 nT/√Hz at 1 mHz and below 100 nT/√Hz at 0.1 mHz; its measurement limit is 600 pT. The MI sensor is characterized by high precision, small size, and low noise, demonstrating considerable potential for application in magnetically sensitive experiments requiring long integration time. This is an effect way to solve the problem that there is on one suitable magnetic sensor at space magnetic field detection, but the sensor requires improvements in temperature stability.

## 1. Introduction

Magnetic sensors based on the magnetoimpedance effect have potential for application in weak magnetic field detection, given their high precision and small size. Since Mohri discovered the magnetoimpedance effect in soft magnetic CoFeSiB amorphous wires [1], the sensors-based magnetoimpedance (MI) effect has been studied widely for over two decades [2,3,4,5,6,7]. This MI effect arises from a combination of a skin effect and a strong field dependence of the circumferential magnetic permeability associated with circular domain wall movements [8]. Magnetic sensors based on the MI effect have been rapidly developed; the latest research shows that MI sensors can reach noise levels below 1 pT/√Hz in the frequency range of 20 to 500 Hz in amorphous wire and a white noise level of 120 pT/√Hz at 2 kHz is obtained in amorphous thin film [9,10], and MI sensors have some advantages in terms of comprehensive strength including small size, sensitivity, linearity, and low power consumption [11]. Despite the high sensitivity being frequently one of the most important properties of MI sensors, low-frequency noise is equally important. Low-frequency noise affects performance, which is directly related to the stability and the measurement limit of magnetic sensors. For anisotropic magneto-resistive (AMR) sensors and flux-gate sensors, their low-frequency noise was recently reported in the sub-millihertz frequencies [12,13]. The noise on commercial magnetic sensors, which ranges from 0.1 Hz to 1 KHz, has been measured in detail [14]. The noise on MI sensors has also been systematically researched [15,16]. However, no previous study conducted research on MI sensors at the millihertz level. The frequency band from mHz to 0.1 Hz is important for space missions, which require long integration time, such as the space gravitational wave detection mission.

Magnetic detection is crucial in many space missions requiring long integration time. For the space gravitational wave detection mission, a real time magnetic field detection within 0.1 mHz–0.1 Hz is strictly required around the test masses [17]. At present, flux-gate magnetometers are popular with scientists due to their high precision and low noise, which were used in the laser interferometer space antenna (LISA) Pathfinder [12,18,19,20]. However, the flux-gate magnetometer has two disadvantages when used in the space gravitational wave detection mission: (1) low spatial resolution due to its large size, and (2) large uncertainty about the magnetic field due to the remnant magnetization of its internal iron core. In previous studies, scientists did not find a better sensor instead of flux-gate magnetometer, so smaller magnetometers with lower remnant magnetization are needed for higher accuracy detection. Diaz-Aguiló developed an AMR sensor for the space gravitational wave detection mission [21]. The AMR sensor measurement results showed that its noise was found to be below 10 nT/√Hz through a combination of two methods: Flipping and electro-magnetic feedback [13,21]. However, inherent 1/f noise (the noise is proportional to the reciprocal of frequency) exists in the AMR sensor [21]. Thus, further studies are needed for using these sensors in the space gravitational wave detection mission. MI sensors have shown excellent comprehensive ability in recent studies; the latest study compared seven aspects of the performance of magnetic sensors [11]. MI sensors are characterized by high precision, small size, and low noise within the family of magnetic sensors. However, we must measure low-frequency noise on MI sensors for testing their ability at sub-millihertz frequencies, which have never been measured prior to this study.

In this study, we evaluated the low-frequency noise and basic performance on a commercial MI sensor (MI-CB-1) from Aichi Steel Co. (Aichi-Ken, Tokai, Japan), from which we removed the AC (alternating current) coupling for applicability at low frequencies. The measurement results show the MI sensor is suitable for magnetically sensitive experiments requiring long integration time. Two problems still exist in the MI sensor: zero drifts and bad temperature stability. The general measurement is outlined below.

## 2. Measurement

**Figure 1:**
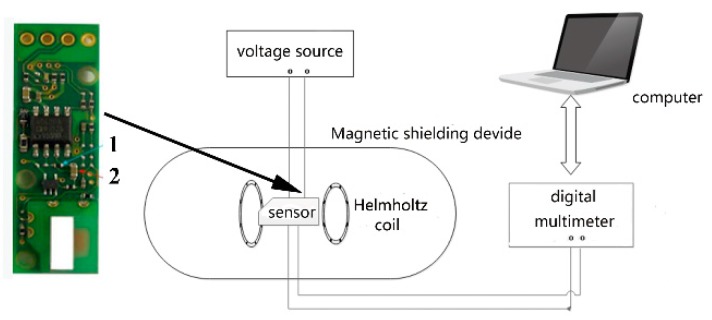
Block diagram of the measurement platform.

The selected MI sensor (MI-CB-1) is an ultra-miniature sensitive device from Aichi Steel Co. (Aichi-Ken, Japan). We created a platform for testing MI sensors, which includes a voltage source, a magnetic shielding device, a Helmholtz coil, a digital multimeter, and a computer. The instruments used in this experiment are low-frequency and high-precision for reducing unwanted noise, and the MI sensor was used in a magnetic shielding device to stabilize the surrounding magnetic field. Figure 1 depicts a diagram of this platform and shows this picture of the MI sensor. We removed the AC coupling through removing this capacitor and short-circuit with lead wire at number 1 and removing this resistor at number 2 for it to work at low frequency. The voltage source (Keysight: E36312A, Santa Rosa, CA, USA) could support the MI sensor and its output was 5 V ± 0.12 mV. A digital multimeter (Keithley: DMM6500, Cleveland, OH, USA) could record the output voltage from the MI sensor with 6_1/2_ significant digits, and the computer and digital multimeter were used for the real time test of the output of the MI sensor. The computer was also needed to analyze the combined data of time and output. A magnetic shield was used to minimize the magnetic field around the MI sensor; it was 37 cm long and 14.4 cm in diameter, with five layers. The magnetic field in the magnetic shielding is less than 3 nT, and the Helmholtz coil produced the desired magnetic field. The performance of the MI sensor was measured by the platform, including its basic performance and low-frequency noise.

The stability and measurement limit of MI sensors depend on low-frequency noise, especially in physics experiments requiring long integration times. Measurement of the MI sensor noise poses some difficulties at low frequency. First, the frequency is so low that the experiment process was over 3 h, during which we ensured the laboratory environment was quiet. Due to the long period, the noise from the devices and ambient temperature were amplified so we could not easily obtain the intrinsic noise from the MI sensor. The low-frequency noise we measured in the laboratory consists of the noise from the MI sensor and other noise, which includes the noise from the laboratory environment, temperature fluctuations, the Helmholtz coil, the voltage source, and the digital multimeter. We used the magnetic shielding to reduce the noise from the laboratory environment to accurately determine the noise from the MI sensor. By measuring the output of the voltage source supplied by the digital multimeter without the MI sensor, we determined that the noise from the voltage source and the digital multimeter is two magnitudes lower than the noise from the MI sensor. The temperature in the laboratory is controlled by an air-conditioner with a temperature variation less than 2 °C. So, the noise data we measured represents the noise from this MI sensor.

## 3. Results

The basic performance of the MI sensor was measured first, including its size, power, sensitivity, linearity, and magnetic field range. Table 1 lists the results of measurement. Despite the high sensitivity, the MI sensor was small, low power, and had high linearity. This MI sensor (11 × 35 × 5 mm) is much smaller than the flux-gate sensor, which has good spatial resolution. Due to simple working principles, small size, and low power, MI sensors can be adapted to various environments and are able to match flux-gate sensors. In space projects, being lightweight, small, highly sensitive, and low power are crucial device properties.

**Figure 2:**
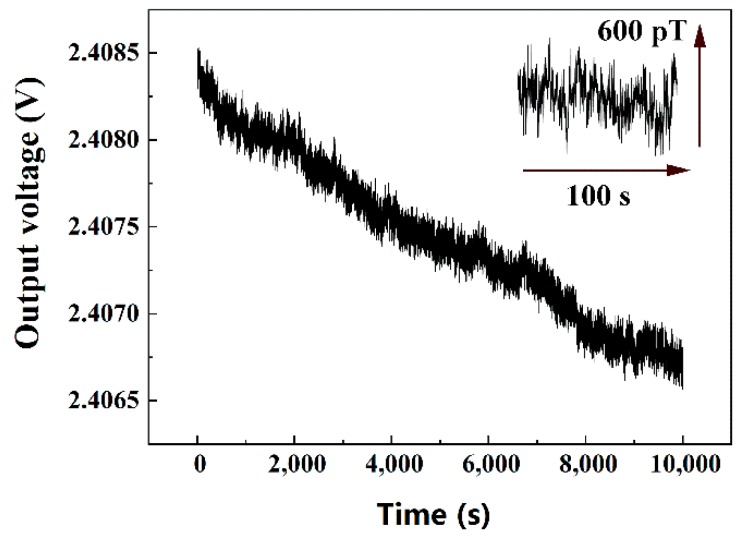
The temporal output voltage evolution without magnetic field (about 2 nT drifts during 10,000 s) and the measurement limit (fluctuations in the output during 100 s).

Low-frequency noise measurement is one of the important methods for evaluating magnetic sensors. After preheating all the instruments for one day, the MI sensor was measured inside magnetic shielding for over three hours. The relationship of the output voltage over time is shown in Figure 2. The line in Figure 2 is sloping downward, which means some zero drifts occurred with the MI sensor. About 2 nT drifts occurred over 10,000 s. The line shows that the real time measurement variation of the MI sensor is less than 600 pT. This excellent measurement limit could compete with that of flux-gate sensors. After measuring the output voltage of the MI sensor for a long time, the low-frequency noise spectral density was obtained after fast Fourier transform (FFT) of the original data, which combined the time and the output of the MI sensor in 0.1 s time intervals and 100,000 data points.

**Figure 3:**
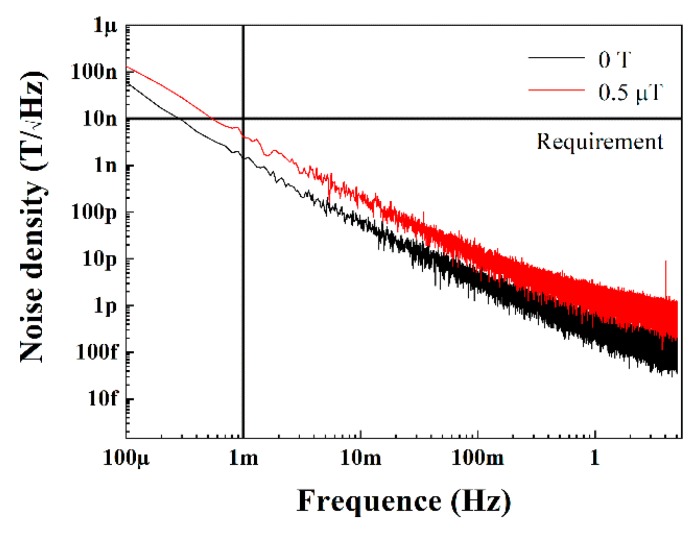
Noise spectral density with and without magnetic field.

**Figure 4:**
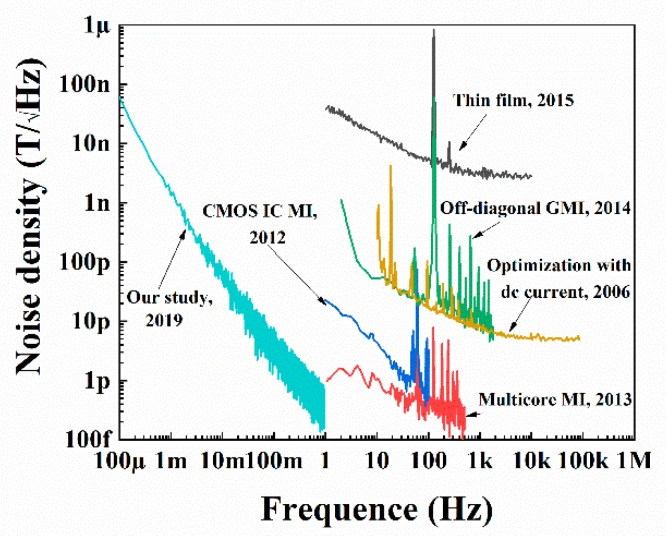
The noises spectral density of various studied sensors, and the notes present the key words and years of these studies.

We measured the low-frequency noise density with and without a direct current (DC) magnetic field (0.5 µT), and each line in Figure 3 is the average of three sets of the data. The low-frequency noise of the MI sensor reached below 10 nT/√Hz above 1 mHz and below 100 nT/√Hz at 0.1 mHz without a DC magnetic field, which represents the real noise background of the MI sensor. We also measured the noise density with a DC magnetic field to determine the noise in the real measurement. The noise background was below 10 nT/√Hz at 1 mHz and slightly above 100 nT/√Hz at 0.1 mHz. The noise with the magnetic field being a little more than the noise without a magnetic field might have occurred due to the noise from the Helmholtz coil. The requirement line in Figure 3 is the requirement of the magnet sensors in the space gravitational wave detection mission [17]. The magnetic field at point L1 is less than 40 nT [18], so this study provides valuable information for the space use of these sensors. The noises of various sensors studies are compared in Figure 4, including the noises of micro-patterned multilayer thin films based GMI Micro sensor (blank line) [10], multicore MI sensor (red line) [9], sensors based on off-diagonal GMI effect (green line) [5], pico-Tesla resolution magneto-impedance sensor based on amorphous wire CMOS IC MI Sensor (deep blue line) [7], sensors with optimization with DC current (yellow line) [16]. Our study offered the noise of MI sensors at ultralow frequency, which previous studies have not measured.

**Figure 5:**
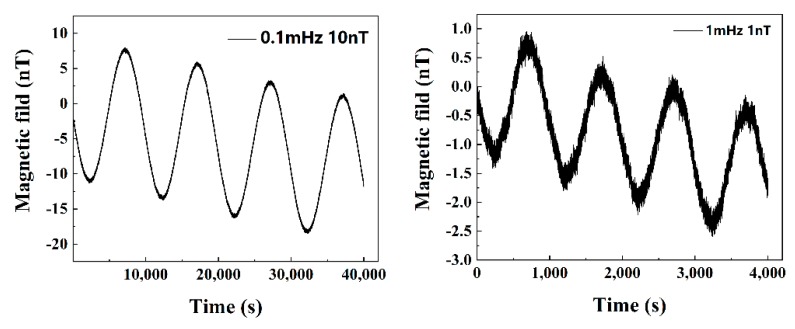
The output voltage at different input magnetic field (**Left**: sinusoidal signal, frequency 0.1 mHz, amplitude 10 nT. **Right**: sinusoidal signal, frequency 1 mHz, amplitude 1 nT).

Figure 5 depicts the output results of the sensor with a sinusoidal wave magnetic field. The Helmholtz coil was employed to produce two sets of magnetic sinusoidal signal: (1) frequency 0.1 mHz and amplitude 10 nT and (2) frequency 1 mHz and amplitude 1 nT, using a signal generator as the power supply. These data were measured by the MI sensor for validating its actual measurement ability. The results in Figure 5 show the tendency of the signal, which means that the MI sensor could easily measure a 1 nT magnetic signal at low frequency. Although the signal is downward sloping, some zero drifts occurred in the MI sensor, which is consistent with the drift shown in Figure 2.

**Figure 6:**
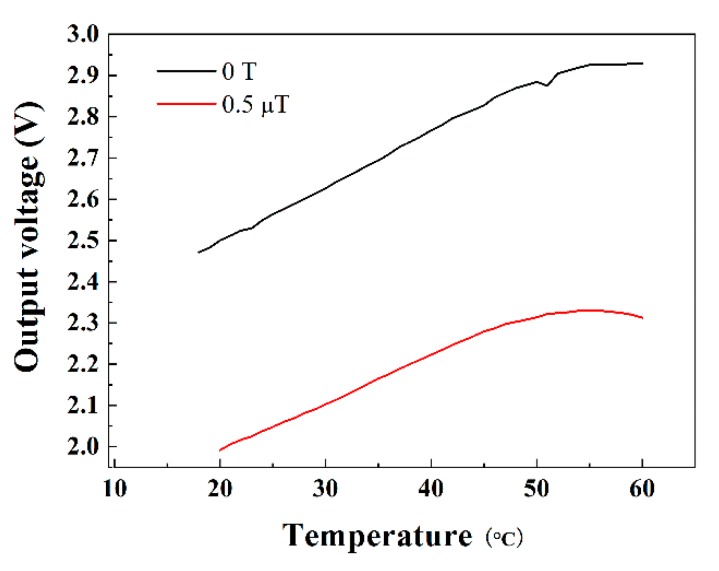
Temperature stability of the output voltage with and without magnetic field.

Finally, we measured the temperature stability of the MI sensor by adding a heating device and a thermometer, and we produced the magnetic field from the heating device vertical to the sensitive direction of the MI sensor, which was less than 4 nT and unchanged. Figure 6 shows the output measured voltage of the MI sensor from 20 to 50 °C with different magnetic fields (0 T and 0.5 µT). The output voltage increases rapidly with temperature, which indicates poor temperature stability (12.47 nT/K) and could only work normally with strictly controlled temperature. Compared with other magnetic sensors, such as the flux-gate sensor (1.2 nT/K) used in the LISA Pathfinder, the temperature stability of MI sensors still requires improvement.

## 4. Conclusions

In this study, for the first time, we measured the low-frequency noise of a commercial MI sensor at sub-millihertz frequencies. Aspects of its basic performance were measured, including its size, power, magnetic field range, linearity, and temperature stability. The results showed that the low-frequency noise of the MI sensor is less than 10 nT/√Hz above 1 mHz and less than 100 nT/√Hz at 0.1 mHz. The MI sensor is characterized by high precision, low power (<100 mW), small size (11 × 35 × 5 mm), and low noise, which was validated by the actual measurement results. However, we observed some zero drifts in the MI sensor and the temperature stability requires further improvement. This commercial MI sensor produces good performance, which means magnetic sensors based on the magnetoimpedance effect show potential for applications requiring long integration time, such as the space gravitational wave mission, and it can efficiently compensate for the shortcomings of flux-gate sensors and AMR sensors. Our study has offered a new way to solve the problem concerning space magnetic field detection. Next, we plan to design a new MI sensor with better temperature stability and lower zero drifts through using better amorphous wires with higher Curie temperature and a redesigned circuit, in which some noise-reduction technologies can be applied [22,23].

## Figures and Tables

**Table 1:** Basic performance of the studied magnetoimpedance (MI) sensor.

Parameters	Value
Linearity	0.16%
Weight	1.195 g
Size	11 × 35 × 5 mm
Input voltage	5 V
Input current	<20 mA
Power	<100 mW
Sensitivity	1241 nT/V
Range	±2 µT
